# Assessment of Public Awareness on Alarming Signs of Posttraumatic Intracranial Hemorrhage in the Jeddah Population: A Cross-Sectional Study

**DOI:** 10.7759/cureus.66613

**Published:** 2024-08-10

**Authors:** Gutaybah S Alqarni, Muhannad M Almutairi, Rayan M Alosaimi, Ahmad A Afandi, Hosam H Alzobaidi, Mujtaba T Al Abdullatif, Essa A Alazmi, Mohammed A Aljunaid

**Affiliations:** 1 Faculty of Medicine, University of Jeddah, Jeddah, SAU; 2 Department of Family and Community Medicine, University of Jeddah, Jeddah, SAU

**Keywords:** symptoms, convulsion, headache, alarming, intracranial hemorrhage, post-traumatic

## Abstract

Background

Post-traumatic intracranial hemorrhage is a life-threatening condition, and early detection and response can significantly reduce morbidity and mortality rates. The aim of this study was to assess public awareness of the alarming signs of intracranial bleeding after trauma in adults in Jeddah, Saudi Arabia.

Methodology

From August 2023 to April 2024, a descriptive cross-sectional study was conducted using a five-scale structured questionnaire: demographics, risk factors for road traffic accidents, alarm signs and symptoms, ability to recognize these signs, and participants who experienced head trauma. The study focused on people aged 18 and over living in Jeddah.

Results

A total of 584 participants were included, with 34.2% males and 65.8% females. Findings revealed that 57% recognized the critical need for medical help after head trauma. Furthermore, only 45% of the population were unaware that low levels of awareness or wakefulness indicate bleeding, reflecting a low awareness level in the adult population. Among children, only 34% recognized changes in eating and lactation habits, and 54% identified continuous crying and irritability. Furthermore, 66% of participants identified loss or change in consciousness as a symptom that required hospital admission, while 60% recognized chronic headaches as a critical sign.

Conclusion

This study revealed the critical need for public health campaigns to improve awareness and understanding of signs of intracranial post-traumatic bleeding. The results highlighted the importance of early medical interventions to reduce the morbidity and mortality associated with this condition.

## Introduction

Intracranial hemorrhage is a life-threatening condition and a leading cause of death. This condition is mainly identified by the site of the bleeding, either between the skull and the brain tissue, which includes epidural, subdural, and subarachnoid bleeding, or within the brain tissue itself, which includes bleeding in the lobes, the pons, the cerebellum, and intraventricular bleeding that occurs in the ventricles. During bleeding, brain tissue undergoes damage since it relies on specific blood vessels to supply oxygen to distinct areas. Importantly, the brain's inability to store oxygen necessitates a continuous blood supply. Any disruption to this supply, such as trauma or ruptured blood vessels, can lead to the accumulation of blood and the formation of hematomas. This hematoma exerts pressure on the surrounding brain tissue, aggravating the injury [1]. Traumatic brain injury (TBI) is the main cause of brain bleeding and contributes greatly to morbidity and mortality. In 2014, TBI was responsible for three million emergency rooms, hospital admissions, and deaths in the United States. During the same year, more than 54,000 adults and about 2,500 children died of TBI [2]. Headache; alterations in consciousness, such as loss or change; double or blurred vision; nausea; vomiting; confusion; and fatigue encompass a spectrum of significant signs and symptoms indicative of TBI. It is crucial to note that the severity of TBI plays an essential role in influencing the presentation and intensity of these manifestations [2,3].

TBI can be diagnosed through a combination of posttraumatic presentations, the nature of the trauma, the Glasgow Coma Scale (GCS), imaging including computed tomography (CT) or magnetic resonance imaging (MRI) scans, and an intracranial pressure monitor. Management of the TBI depends on the severity of the injury. In cases of mild injuries, treatment may include rest and analgesics to relieve headaches. However, in moderate to severe cases, patients may require oxygen support, blood supply management, blood pressure maintenance, and prevention of further head and neck injuries. These cases often involve entering an intensive care unit to minimize secondary damage, along with the administration of medications such as anti-seizure drugs, coma-inducing agents, and diuretics. Additionally, emergency surgery might be necessary to remove hematomas and prevent additional harm to brain tissue [4]. Late detection of posttraumatic brain injury and late medical seeking can lead to several complications including a vegetative state, coma, and brain death [5].

Few studies have investigated public awareness of the symptoms of intracranial posttraumatic hemorrhage. In 2019, two studies were conducted in Jeddah to evaluate public awareness of the alarming signs of post-traumatic intracranial hemorrhage; both concluded that there is a need to raise public awareness. Identifying the alarming signs and seeking medical attention is crucial [6,7]. Moreover, this study aimed to scientifically evaluate the public awareness of the alarming signs of intracranial posttraumatic hemorrhage among the Jeddah population.

## Materials and methods

Design, participants, and setting

This descriptive cross-sectional study was conducted from August 2023 to April 2024. We included all participants aged 18 years and older living in Jeddah, Saudi Arabia, to explore their awareness of the risk of posttraumatic intracranial hemorrhage.

Sample size

The Raosoft sample size calculator (http://www.raosoft.com/samplesize.html) was used to calculate the sample size. With a margin of error of 5%, a confidence interval of 95%, and a response distribution of 50%, the minimal sample size was determined to be 385. Our study included 584.

Questionnaire 

With permission, we used a validated and published questionnaire [6]. This questionnaire comprised five scales: demographic information, risk factors of road traffic accidents (RTA), awareness of alarming signs and symptoms of posttraumatic intracranial hemorrhage, the ability to recognize these alarming signs and symptoms, and prior experience with head trauma among participants.

Data collection and statistical analysis

Data collection and statistical analysis: Data collection and management are carried out using Google Forms (Google LLC, Mountain View, CA) and Microsoft Office Excel (Microsoft Corp., Redmond, WA) The categories are presented in tables and figures in the form of frequencies and percentages. The statistical significance of the P value was defined as ≤0.05, with a 95% confidence interval. Statistical analysis was done using Statistical Product and Service Solutions (SPSS, version 29; IBM Corporation, Armonk, NY).

Ethical consideration

The study was approved by the Bioethics Committee of Scientific and Medical Research, Directorate of Health Affairs, Ministry of Health (MOH), Jeddah (#A01742). The questionnaire began with a written consent agreement indicating that they had signed their consent to participate in the study by submitting the questionnaire.

## Results

Table 1 summarizes the demographic data of 584 participants. The study included 34.2% males and 65.8% females, with the majority being within the 18-30 age group. Most participants (81.3%) had a high degree of education. The participants' occupations varied, with 5.8% being health practitioners, 24.3% other employees, and 50.0% being students. Furthermore, 76.9% had previously dealt with children, with relationships ranging from mothers (14.2%) to family members (56.3%) and work-related interactions (6.3%).

**Table 1:** Demographic characteristics of the participants.

Characteristics		Count (%)
Gender	Male	200 (34.2%)
	Female	384 (65.8%)
Age group	18-30	415 (71.1%)
	31-40	79 (13.5%)
	41-50	57 (9.8%)
	>50	33 (5.7%)
Number of family members	2-4	123 (21.1%)
	5-8	380 (65.1%)
	>8	81 (13.9%)
Are there children in the family under the age of 15?	No	197 (33.7%)
	Yes	387 (66.3%)
Elderly over 60 years old	No	322 (55.1%)
	Yes	262 (44.9%)
Marital status	Single	395 (67.6%)
	Married	170 (29.1%)
	Divorced	14 (2.4%)
	Widow	5 (0.9%)
Educational level	Uneducated	2 (0.3%)
	Educated but did not have secondary school certificate (primary and intermediate)	8 (1.4%)
	Secondary school certificate	99 (17.0%)
	High educational level	475 (81.3%)
Carrier	Employee (health practitioner)	34 (5.8%)
	employee (other)	142 (24.3%)
	Student (medicine and surgery)	140 (24.0%)
	Student (other specialty)	152 (26.0%)
	unemployed	116 (19.9%)
Do you deal with children frequently?	No	135 (23.1%)
	Yes	449 (76.9%)
If your answer was yes, what is the type of your relationship with the children?	Mother	83 (14.2%)
	A family member other than the mother	329 (56.4%)
	At work, for example: teaching, nursing, etc.	37 (6.3%)
	I don’t deal with children frequently	135 (23.1%)

Table 2 shows the participant’s awareness of the risk factors associated with head trauma. Most participants reported not having hypertension (94%), not receiving blood-thinning medication (96.4%), having blood disorders such as hemophilia (98%), or having chronic hepatitis (99.3%). A significant proportion of participants demonstrated awareness that these conditions might increase the risk of intracranial hemorrhage after head trauma, with 40% of the participants acknowledging the risk factors related to hypertension, blood-thinning medications, blood disorders, and chronic hepatitis. Furthermore, most of the participants (59.6%) confirmed that repetitive simple head trauma can cause intracranial hemorrhage. In addition, 57% of respondents acknowledged that it is critical to obtain medical help after experiencing a fit following head trauma.

**Table 2:** Participants’ awareness of risk factors.

Second scale questions		Count (Percent)
Do you have HTN?	No	549 (94.0%)
	Yes	35 (6.0%)
Do you use blood-thinning medications?	No	563 (96.4%)
	Yes	21 (3.6%)
Do you have any diseases that clot blood, such as hemophilia?	No	575 (98.5%)
	Yes	9 (1.5%)
Do you have chronic hepatitis?	No	580 (99.3%)
	Yes	4 (0.7%)
Do you know that what we mentioned in previous questions (HTN, blood-thinning medications, blood disorders, such as hemophilia, and chronic hepatitis) makes you more vulnerable to having ICH after head trauma?	No	350 (59.9%)
	Yes	234 (40.1%)
Do you know that repetitive simple head trauma for an old age person may cause ICH for him \ or her?	No	236 (40.4%)
	Yes	348 (59.6%)
Do you know that after you awake from your fit after head trauma you must go to the hospital? (As it is a lucid interval, you may experience another fit after this period)?	No	251 (43.0%)
	Yes	333 (57.0%)

Table 3 shows the participant’s awareness of the alarming symptoms that required admission to the hospital. Sixty-six percent of participants recognized loss or change in consciousness; 60% identified chronic headaches as alarming; 55% were aware of signs that indicate low levels of awareness or wakefulness; 61% recognized nausea or vomiting; and changes in vision, convulsions, and clear fluid from the nose or ears were reported by 61%, 67%, and 43% of participants, respectively. Sixty-nine percent recognized neurological signs and symptoms as alarming, while only 25.9% knew not to drink until CT results were reassured when they felt thirsty after head trauma.

**Table 3:** Awareness of participants regarding alarming symptoms that required admission to the hospital.

Third scale questions		Count (%)
Loss or change in conciseness within minutes to hours post-head trauma?	I do not know	195 (33.4%)
	I know	389 (66.6%)
Chronic headache?	I do not know	230 (39.4%)
	I know	354 (60.6%)
Decrease the level of awareness or wakefulness (e.g., fatigability, difficulty in waking up from sleep)?	I do not know	259 (44.3%)
	I know	325 (55.7%)
Nausea or vomiting?	I do not know	223 (38.2%)
	I know	361 (61.8%)
Changes in vision as double vision or dilatation of one pupil?	I do not know	228 (39.0%)
	I know	356 (61.0%)
Convulsion?	I do not know	190 (32.5%)
	I know	394 (67.5%)
Clear fluid coming out from the nose or ears?	I do not know	329 (56.3%)
	I know	255 (43.7%)
Neurological signs and symptoms include slurred speech, weakness of the arms, legs, or face, and loss of balance.	I do not know	176 (30.1%)
	I know	408 (69.9%)
Do you know that when you feel thirsty after head trauma you should not drink until the CT result is reassured?	I do not know	433 (74.1%)
	I know	151 (25.9%)

Table 4 demonstrates the awareness and ability to recognize alarming signs and symptoms in children. A total of 34% recognized changes in eating and lactation habits, 54% identified continuous crying and irritability, 50% were aware of changes in concentration and interest, 63% recognized inability to balance and unbalanced walking, 55% acknowledged vomiting and fatigability, and only 38% identified changes or decreases in academic performance.

**Table 4:** Awareness and ability to recognize alarming signs and symptoms in children.

Forth scale questions		Count (%)
Change in eating and lactation habit	I do not know	382 (65.4%)
	I know	202 (34.6%)
Continuous crying and irritability	I do not know	266 (45.5%)
	I know	318 (54.5%)
Change in the ability of concentration, decreased interest in preferred games and activities	I do not know	290 (49.7%)
	I know	294 (50.3%)
Change in sleeping pattern?	I do not know	353 (60.4%)
	I know	231 (39.6%)
Loss of acquired primitive skills, for example, loss of ability to control urination and toilet use	I do not know	312 (53.4%)
	I know	272 (46.6%)
Inability to balance, unbalanced walking	I do not know	215 (36.8%)
	I know	369 (63.2%)
Vomiting and fatigability academic performance	I do not know	258 (44.2%)
	I know	326 (55.8%)
Change or decrease in academic performance	I do not know	360 (61.6%)
	I know	224 (38.4%)

Table 5 demonstrates participants' responses to previous head trauma experiences. In the event of head injury, 33.2% would go to the hospital immediately, 14.6% would base their decision on severity alone, 48.5% would consider both severity and symptoms, and 41.8% reported having experienced previous head trauma. A total of 50.2% of the participants who experienced head trauma rated the severity as 1-3/10, 38% rated it as 4-7/10, and 11.8% rated it as 7-10/10. Furthermore, 74.5% reported no loss of consciousness, with only 16.6% seeking hospital care after head trauma. Additionally, 56.7% of the participants reported no associated symptoms, 35.2% reported symptoms mentioned in the questionnaire, and 8.2% reported other symptoms.

**Table 5:** Previous history of head trauma.

Fifth scale questions		Count (%)
When you experience head trauma what will be your action?	I will go to the hospital immediately	194 (33.2%)
	Depending on the severity of the injury, regardless of symptoms	85 (14.6%)
	Depending on the severity and symptoms	283 (48.5%)
	Others	22 (3.8%)
Did you have previous head trauma, for example, a fall, a blow, or an accident? etc.?	No	340 (58.2%)
	Yes	244 (41.8%)
If yes, please answer the following: Assessing the severity of 10	1-3/10	122 (50.2%)
	4-7/10	93 (38.0%)
	7-10\10	29 (11.8%)
Did the trauma associate with loss of consciousness?	No	182 (74.5%)
	Yes	62 (25.5%)
If your answer was yes, did you go to the hospital?	No	19 (7.9%)
	Yes	41 (16.6%)
	I experienced head trauma without loss of consciousness	184 (75.5%)
Did the trauma associate with any symptoms that had been mentioned above on the questionnaire?	No	138 (56.7%)
	Yes	86 (35.2%)
	Other symptoms	20 (8.2%)

Table 6 provides a comparison of participants' awareness of alarming symptoms between those who had experienced head trauma and those who did not. The awareness levels were similar for most symptoms and did not significantly differ. However, there was notably a greater awareness of decreased awareness or wakefulness among those who had experienced head trauma. Furthermore, individuals who had not had a head injury also showed a greater awareness of waiting to drink until CT results were confirmed. These results indicate generally comparable awareness levels between the two groups, with some minor differences in certain symptoms.

**Table 6:** Comparison of the awareness of participants regarding alarming symptoms that require presentation to the hospital between participants who experienced head trauma and participants who did not.

Parameter		Did you have previous head trauma, for example, a fall, blow, or accident? etc.?		P-value
		No	Yes	
		Count (%)	Count (%)	
Loss or change in conciseness within minutes to hours post-head trauma?	I do not know	113 (33.2%)	82 (33.6%)	0.925
	I know	227 (66.8%)	162 (66.4%)	
Chronic headache?	I do not know	138 (40.6%)	92 (37.7%)	0.482
	I know	202 (59.4%)	152 (62.3%)	
Decrease the level of awareness or wakefulness (e.g., fatigability, difficulty in waking up from sleep)?	I do not know	162 (47.6%)	97 (39.8%)	0.058
	I know	178 (52.4%)	147 (60.2%)	
Nausea or vomiting?	I do not know	135 (39.7%)	88 (36.1%)	0.372
	I know	205 (60.3%)	156 (63.9%)	
Changes in vision as double vision or dilatation of one pupil?	I do not know	134 (39.4%)	94 (38.5%)	0.828
	I know	206 (60.6%)	150 (61.5%)	
Convulsion?	I do not know	108 (31.8%)	82 (33.6%)	0.639
	I know	232 (68.2%)	162 (66.4%)	
Clear fluid coming out from the nose or ears?	I do not know	197 (57.9%)	132 (54.1%)	0.356
	I know	143 (42.1%)	112 (45.9%)	
Neurological signs and symptoms such as slurred speech, weakness of the arms, legs, or face, and loss of balance?	I do not know	110 (32.4%)	66 (27.0%)	0.168
	I know	230 (67.6%)	178 (73.0%)	
Do you know that when you feel thirsty after head trauma you should not drink until the CT result is reassured?	I do not know	264 (77.6%)	169 (69.3%)	0.022
	I know	76 (22.4%)	75 (30.7%)	

## Discussion

Intracranial bleeding is a serious and potentially deadly condition that occurs as a result of bleeding inside the skull or brain. Epidural hemorrhage occurs between the skull and the dura mater; its symptoms include severe headache, loss of consciousness, and rapid neurological deterioration, which requires immediate surgical treatment for drainage of the hematoma and to relieve pressure on the brain. The symptoms in subdural bleeding, which happens between the dura mater and the arachnoid membrane, may have a more gradual onset compared with epidural bleeding, and treatment will usually depend on the size of the hematoma and the presence of symptoms. Subarachnoid hemorrhage is one of the forms of bleeding in the subarachnoid space between the arachnoid membrane and the pia mater, with symptoms such as a sudden severe "thunderclap" headache and decreased level of consciousness, that needs to be diagnosed on time and treated to prevent complications such as vasospasm and hydrocephalus, or which could take place in the brain tissue itself, including the lobes and cerebellum [8-10]. TBI is a major cause, marked by symptoms such as headaches, changes in consciousness, visual disturbances, nausea, and fatigue, with the severity of TBIs determining the intensity of these symptoms. Diagnosis combines clinical assessment, GCS, imaging tests, and monitoring of intracranial pressure. Treatment varies with injury severity, from rest and pain relief for mild cases to oxygen support, blood pressure management, and potentially surgery for moderate to severe injuries, aiming to prevent further damage and complications such as coma or brain death [1,3-5]. This highlights the importance of public awareness and education on recognizing early symptoms and understanding the risks that can lead to timely medical intervention, significantly lowering the chance of severe complications. This cross-sectional study aimed to assess the level of public awareness regarding the alarming signs of posttraumatic intracranial hemorrhage among the Jeddah population. Our findings reveal significant insights into the communities’ knowledge of and preparedness for responding to head trauma incidents. The demographic data indicated a predominance of younger, highly educated participants, with a notable representation of students (50.0%). This demographic distribution is crucial because it suggests that the majority of our respondents are from a segment of the population that is likely active and potentially at greater risk of experiencing or witnessing head trauma incidents.

Awareness of risk factors and symptoms associated with intracranial hemorrhage showed variability among participants. A substantial portion did not recognize the importance of hypertension, blood-thinning medications, blood disorders, chronic hepatitis, or repetitive head trauma as risk factors. However, a considerable knowledge gap exists in recognizing risk factors, which is concerning given their potential to exacerbate traumatic outcomes. Our findings are consistent with the literature, which also identified a notably low awareness of risk factors [6,7]. Our study revealed that severe symptoms necessitating hospital presentation indicate a relatively high awareness of neurological symptoms, loss of consciousness, and convulsions. However, there is low awareness regarding the precaution of not drinking until CT results and clear fluid coming out from the nose or ears. These findings are in line with both studies carried out by Mohammed et al. and Mohammed Alnefai et al. This knowledge illustrates a critical gap in the awareness of the public's emergency response [6,7]. This finding is particularly alarming, as it could potentially affect patient outcomes negatively. The literature suggests that early and appropriate responses to head trauma can significantly influence the prognosis of intracranial hemorrhage patients [11].

The awareness and ability to recognize alarming signs and symptoms in children presented mixed results, with a lower recognition of changes in eating and lactation habits and changes in sleeping patterns. These findings are consistent with those of Mohammed et al. and Mohammed Alnefai et al. [6,7]. This finding highlights the need for targeted educational programs that address the unique symptoms of head trauma in children. A third of the participants would immediately seek hospital care following head trauma, and more than half considered both the severity of the injury and the symptoms before deciding to go to the hospital. This disparity was also found in a similar study done by Mohammed et al. [6]. General awareness of the alarming symptoms such as loss of consciousness, chronic headache, and nausea or vomiting, was relatively high among both groups. Participants with previous head trauma showed slightly higher awareness of decreased wakefulness, which may reflect their personal experiences. However, both groups showed a lack of awareness of the recommendation not to drink until a CT scan is clear, suggesting a gap in knowledge.

While this study sheds light on these vital issues, we acknowledge the limitations including self-reported data and geographic limitations as focusing on the Jeddah population limits the generalizability of the findings to other regions within Saudi Arabia. Future research should address these by including a more diverse sampling method and using objective measures of awareness. Additionally, investigating the long-term impact of head trauma on individual awareness and behavior could help improve intervention methods and overall health outcomes.

## Conclusions

This study aimed to measure the population’s awareness of the alarming signs and symptoms associated with head trauma. Although some symptoms, such as neurological signs, loss of consciousness, and convulsions, are well recognized, gaps in awareness are evident in terms of risk factors and less obvious symptoms, such as clear liquid discharge from the nose or ears. These conclusions emphasize the need for comprehensive public health education campaigns to improve the ability of the general population to respond effectively to head injuries, potentially reducing the morbidity and mortality associated with intracranial bleeding.
